# IL-23促进人肺腺癌A549细胞的迁移和侵袭

**DOI:** 10.3779/j.issn.1009-3419.2012.05.01

**Published:** 2012-05-20

**Authors:** 森 张, 军 黎, 洁 张, 乐 张, 苹 林

**Affiliations:** 610041 成都，四川大学华西医院生物治疗国家重点实验室老年医学研究室 Division of Geriatrics, State Key Laboratory of Biotherapy, West China Hospital, Sichuan University, Chengdu 610041, China

**Keywords:** IL-23, A549肺腺癌细胞, 迁移, 侵袭, MMP-9, IL-23, Lung adenocarcinoma cells A549, Migration, Invasion, MMP-9

## Abstract

**背景与目的:**

前炎症因子白细胞介素23（interleukin 23, IL-23）是与慢性炎症和肿瘤微环境相关的一种重要的细胞因子，同时IL-23受体在结直肠癌、肺癌、口腔鳞癌等肿瘤细胞中有表达。本研究旨在探讨IL-23能否促进人肺腺癌细胞A549的迁移和侵袭并探讨其机制。

**方法:**

用划痕试验和Transwell小室法测定IL-23对A549的迁移和侵袭的影响，用Real-time PCR和ELISA检测IL-23对基质金属蛋白酶9（matrix metalloproteinase 9, MMP-9）的mRNA和蛋白表达的影响，通过IL-23中和抗体阻断IL-23的作用，进一步证实IL-23对A549迁移和侵袭的影响。

**结果:**

IL-23明显增加了A549细胞的迁移和侵袭能力；同时IL-23能提高A549细胞MMP-9的mRNA表达和其培养上清中MMP-9的蛋白表达，IL-23中和抗体能有效地阻断IL-23对A549的迁移和侵袭的作用。

**结论:**

IL-23可刺激A549细胞表达MMP-9，从而促进A549细胞的迁移和侵袭。

## Abstract (en)

**Background and objective:**

Interleukin 23 (IL-23) is a pro-inflammatory cytokine that plays an important role in inflammatory disease and tumor microenvironment. The IL-23 receptor is expressed in colon, lung, and oral carcinomas. We performed this study to investigate whether IL-23 promotes directly carcinoma cell migration and invasion as well as further explore its mechanism.

**Methods:**

The migration and invasion of human lung adenocarcinoma A549 cells induced by IL-23 were detected by a scratch test and Transwell experiment. MMP-9 expression of the mRNA and protein levels of A549 cells cultured with and without IL-23 was respectively detected by Real-time PCR and ELISA. The effect of IL-23 on A549 cells was further verified using anti-IL-23p19 neutralization antibody (Ab IL-23p19) to eliminate IL-23.

**Results:**

IL-23 remarkably promoted A549 cell migration and invasion. MMP-9 expression in A549 cells was upregulated by IL-23 stimulation. In addition, the effect of IL-23 on the migration and invasion of A549, as well as the MMP-9 expression in A549 cells induced by IL-23, was eliminated by Ab IL-23p19.

**Conclusion:**

IL-23 promotes the migration and invasion of A549 cells by inducing MMP-9 expression.

许多肿瘤的发生都与慢性炎症性疾病相关^[1]^，例如：结肠癌、非小细胞肺癌、胃癌等^[2-4]^。白细胞介素23（interleukin 23, IL-23）是近年发现的一种与慢性炎症相关的重要的细胞因子^[5]^；目前研究^[6]^认为IL-23通过促进TH17细胞的作用引起炎症，从而促进肿瘤的发生发展；*IL-23*基因敲除的小鼠有通过增加T细胞渗透到病变组织抵抗化学物引起的致癌作用^[5]^。

IL-23通过与IL-23受体（IL-23 receptor, IL-23R）结合而发挥其生物学功能。IL-23R是由IL-12Rβ1和IL-23R两个亚基组成的膜受体^[7]^。IL-23R主要在DC细胞、巨噬细胞和T细胞中表达^[8]^，最近有研究^[9, 10]^报道IL-23R在肿瘤细胞表达并且有促进肿瘤细胞增殖的作用。肿瘤的侵袭和迁移能力是判断肿瘤恶性程度的一个重要指标，与肿瘤的发生、发展息息相关，而目前尚未见有关IL-23直接影响肿瘤细胞迁移和侵袭力的报道。据此，本研究旨在探索IL-23是否可以直接促进肿瘤细胞的迁移和侵袭。

## 1 材料与方法

### 1.1 细胞培养及处理

人肺腺癌细胞株A549常规培养于含10%FCS、100 IU/mL青霉素和100 μg/mL链霉素的RPMI-1640培养液（Gibco）中，于37 ℃、5%CO_2_的饱和湿度孵箱中条件下培养。将对数生长期的A459细胞以5×10^5^个/孔接种于6孔板上、摇匀，培养24 h；经预实验最终确定加入IL-23的浓度为10 ng/mL（R & D Systems），4 h后提取RNA，用于Real-time PCR检测；24 h后收集培养上清，离心去除杂质，放入-20 ℃保存用于ELISA检测。根据eBioscience公司说明书，抗IL-23的中和抗体Ab IL-23p19（eBioscience）按0.3 μg/mL中和0.2 ng/mL IL-23作用加入进行中和IL-23的作用实验。

### 1.2 HE染色

肺腺癌切片常规采用HE染色。显微镜下观察肿瘤组织中肿瘤细胞及其微环境中炎性细胞浸润情况。

### 1.3 划痕实验测定细胞迁移

将对数生长期的A549细胞以5×10^5^个/孔接种于6孔板中，培养24 h后，用无菌的吸头沿培养孔的正中线刮一道直线形划痕，0.01 mol/L PBS冲洗游离细胞，加入IL-23（10 ng/mL）72 h后于显微镜下观察拍照，并计数刮痕内迁移的细胞数量，本实验重复3次。

### 1.4 Transwell小室法测定侵袭力

将铺有基质胶的Transwell小室（Chemicon USA）置于24孔板，在小室外加入500 μL含10%FBS的RPMI-1640完全培养液，小室内加入300 μL无血清培养基重悬的肿瘤细胞液（1×10^6^个/mL），培养箱中培养72 h之后取出Transwell小室，PBS清洗，用棉签擦去微孔膜上层的基质胶及细胞，95%乙醇固定10 min，结晶紫染色10 min，PBS洗3遍，自然风干后，用30%乙酸溶解结晶，将溶液吸入96孔板中，于560 nm测吸光度。此实验重复3次。

### 1.5 Real-time PCR检测基质金属蛋白酶9（matrix metalloproteinase 9, MMP-9）的表达

用TAKARA试剂盒从1×10个/mL细胞提取总RNA，用逆转录试剂盒合成cDNAs，用iCycler iQ Real-time PCR Detection System（Bio-Rad240PuHercules, CA, USA）检测cDNAs（等于40 ng的总RNA）特异性引物：MMP-9上游引物：GCC TTT GGA CAC GCA CG；MMP-9下游引物：AGC GGT CCT GGC AGA AAT AG。GAPDH上游引物：ACC ACA GTC CAT GCC ATC AC；GAPDH下游引物：TCC ACC ACC CTG TTG CTG TA。每个样本重复3次，每个样本的每个基因相对量以内参GAPDH标准化。用SYBR Premix Ex Taq Ⅱ kit检测目的产物，按照如下条件进行实时PCR反应：（95 ℃、15 s；60 ℃、45 s）×40个循环。用Gene Expression Macro（version 1.1）software（Bio-Rad）计算相对表达值。

### 1.6 ELISA检测细胞上清MMP-9的水平

6孔板每孔铺5×10^5^个/mL A549细胞，加入IL-23（10 ng/mL）分别作用0 h、0.5 h、1 h、2 h、4 h、8 h和24 h，收集每个时间段的细胞培养上清用于ELISA。根据ELISA试剂盒（博士徳）检测细胞上清MMP-9，加入终止液后，酶标仪上根据待测样本的吸光度值（450 nm）经标准曲线查得样本的MMP-9浓度。

### 1.7 统计学分析

采用SPSS 11.5统计软件进行分析。3次独立实验的数据采用Mean±SD表示，实验组与对照组的组间差异比较采用*t*检验分析，*P* < 0.05为差异具有统计学意义。

## 2 结果

### 2.1 肺腺癌组织周围有大量炎性细胞浸润

人肺腺癌组织HE染色（图 1）可见腺癌组织周围有大量的淋巴细胞浸润。

**1:**
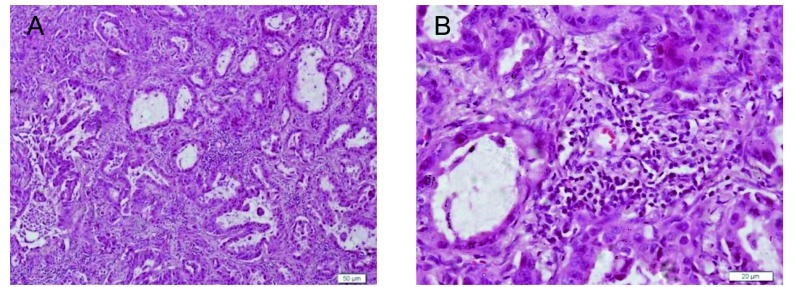
人肺腺癌组织HE染色。A：×100；B：×400。 Adenocarcinoma of human lung was stained by hematoxylin and eosin. A: ×100; B: ×400.

### 2.2 IL-23促进A549细胞的迁移和侵袭力

划痕实验（图 2）结果显示，加入IL-23组的A549细胞（IL-23组）在72 h迁移细胞数明显多于PBS组，两组迁移细胞数有明显差异（*P* < 0.05）。侵袭实验（图 3）显示IL-23组A549细胞穿过人工基底膜的细胞数（160个）及溶解结晶后测得的吸光度（0.712）明显高于PBS处理组的细胞数（50个）及吸光度（0.302），两组处理有明显差异（*P* < 0.01）。

**2:**
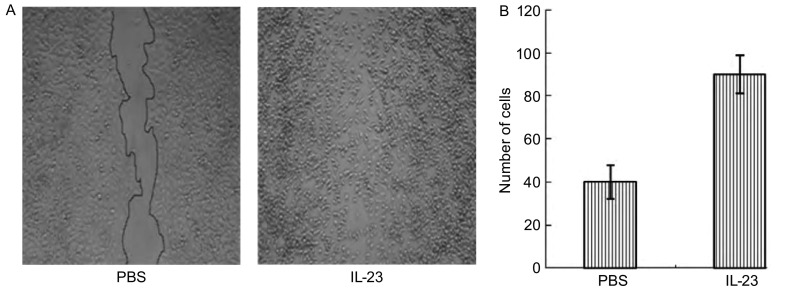
IL-23增加A549的迁移。A：用PBS和IL-23分别处理72 h后A549迁移（×100）；B：划痕区迁移细胞数。 IL-23 promoted the mobility of A549. A: A549 cells was treated with PBS or IL-23 (10 ng/mL) for 72 h (×100); B: The number of mobile A549 cells counted.

**3:**
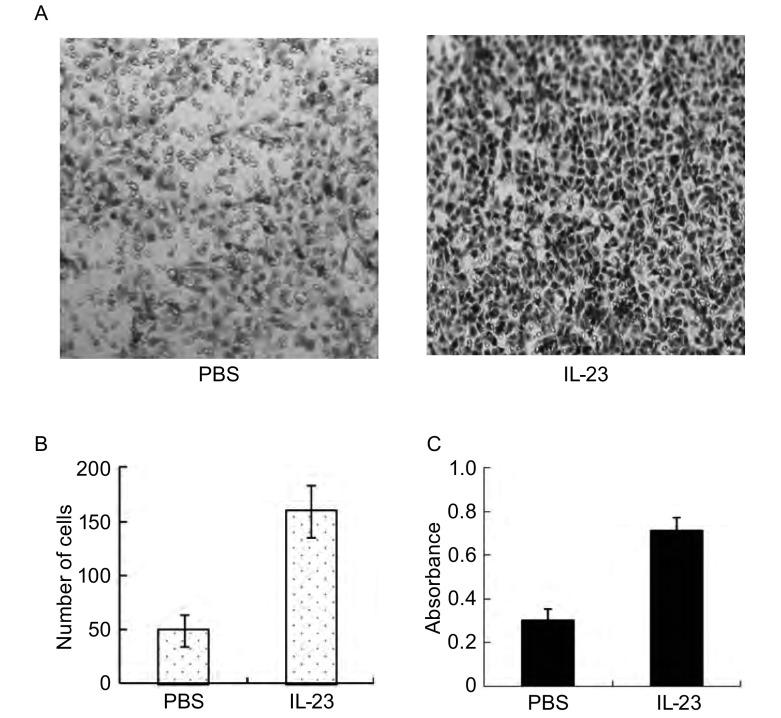
IL-23增加A549的侵袭力。A：分别用PBS、IL-23处理后侵袭细胞结晶紫染色（×200）；B：侵袭细胞数；C：侵袭细胞结晶紫染色吸光值。 IL-23 promoted the invasion of A549. A: A549 cells was treated with PBS or IL-23 (10 ng/mL) and invading A549 was stained by crystal violet (×200; B: The number of invading cells; C: The absorbance value of invading.

### 2.3 IL-23中和抗体抑制IL-23促进A549细胞的迁移和侵袭力的作用

Ab IL-23p19是针对IL-23p19亚基的鼠源性单克隆抗体，可通过结合p19亚基而中和IL-23的生物学作用。划痕实验（图 4）结果显示，加入IL-23+Ab IL-23p19组的A549细胞在72 h后，迁移细胞数明显少于加入IL-23但未加Ab IL-23p19组，两组具有统计学差异（*P* < 0.05）。侵袭实验（图 5A、图 5B、图 5C）显示IL-23+Ab IL-23p19处理组穿过人工基底膜细胞的细胞数和吸光值明显少于IgG+IL-23处理组，两组有明显的差异（*P* < 0.01）。

**4:**
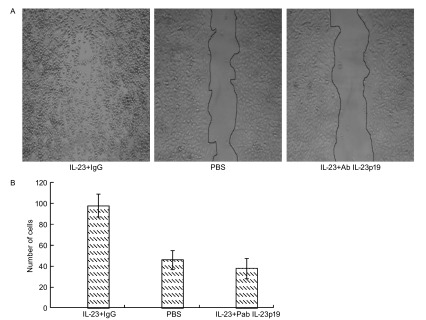
IL-23的中和抗体阻断IL-23对A549迁移和侵袭力的作用。A：用IL-23+IgG、PBS、IL-23+Ab IL-23p19分别处理72 h后A549细胞迁移（×100）；B：划痕区迁移细胞数。 Ab IL-23p19 blocked the effect of IL-23. A: A549 cells was treated with IL-23+IgG, PBS and IL-23+Ab IL-23p19 for 72 h (×100); B: The number of mobile A549 cells counted.

**5:**
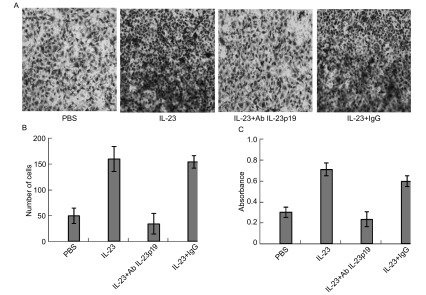
IL-23的中和抗体阻断IL-23对A549侵袭力的作用。A：采用结晶紫染色观察PBS、IL-23、IL-23+Ab IL-23p19、IL-23+IgG处理后A549细胞的侵袭能力（×200）；B：侵袭细胞数；C：侵袭细胞结晶紫染色吸光值。 Ab IL-23p19 blocked the effect of IL-23. A: A549 cells was treated with PBS, IL-23, IL-23+Ab IL-23p19 and IL-23+IgG and invading A549 was stained by crystal violet (×200); B: The number of invading cells; C: The absorbance value of invading.

### 2.4 IL-23增加MMP-9 mRNA的表达并促进细胞分泌MMP-9

A549细胞在IL-23作用0 h、0.5 h、1 h、2 h、4 h、8 h、24 h后，4 h时IL-23处理的细胞中MMP-9的mRNA表达明显增加约2倍，之后达到平台期（图 6A），上清分泌的MMP-9蛋白呈直线增加，24 h后增加约为0 h的2.5倍（图 6B）。

**6:**
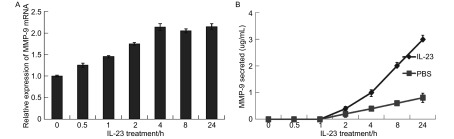
IL-23刺激A549细胞MMP-9表达。A：Real-time PCR测定MMP-9 mRNA表达；B：ELISA检测A549细胞培养上清MMP-9表达。 IL-23 improved MMP-9 expression of A549 cells. A: Expression of MMP-9 mRNA was measured by Real-time PCR; B: MMP-9 in the supernatant of A549 was detected by ELISA.

### 2.5 IL-23中和抗体抑制IL-23的作用

A549细胞分别用IL-23+Ab IL-23p19、IL-23+IgG、IL-23处理，IL-23+Ab IL-23p19处理组4 h时*MMP-9*基因表达明显低于IL-23+IgG组和IL-23组（图 7A），且处理24 h时分泌的MMP-9蛋白明显减少（图 7B）。

**7:**
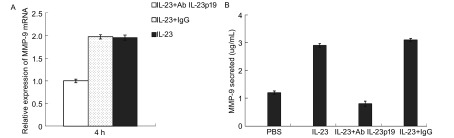
中和抗体IL-23阻断IL-23促进MMP-9表达的作用。IL-23或IL-23+Ab IL-23p19处理A549细胞，鼠的IgG作阴性对照。A：Real-time PCR测定MMP-9 mRNA表达；B：ELISA检测A549细胞培养上清MMP-9表达。 Ab IL-23p19 blocked the effect of IL-23 on MMP-9 expression. A549 were treated with IL-23 alone or IL-23 and Ab IL-23p19. The mouse IgG was used as a negative control to instead Ab IL-23p19. A: Expression of MMP-9 mRNA was measured by Real-time PCR; B: MMP-9 in the supernatant of A549 was detected by ELISA.

## 3 讨论

许多肿瘤与慢性炎症相关，在慢性炎症的部位很多肿瘤的发生率明显上升，长期用抗炎药可以减少肿瘤发生率^[11]^。IL-23是近年来发现的一种同时参与慢性炎症形成和肿瘤发生的细胞因子^[5]^；它是IL-12大家族的一员，与IL-12共有p40亚基，同时又具有自己特异性的亚基p19^[12]^。目前也有见IL-23直接促进肿瘤增殖的报道^[10]^。

肿瘤细胞的侵袭与迁移是指肿瘤细胞通过组织或血管的基底膜种植到原发病灶以外的过程，是肿瘤转移必不可少的一个过程，它将直接影响肿瘤患者的预后，而许多细胞因子也与肿瘤的迁移和侵袭相关。如IL-12可以通过NK和/或自然杀伤性T细胞抑制小鼠肝癌的转移^[13]^；IL-17A可以通过NF-κB上调MMP-2和MMP-9的表达来促进肝癌细胞的侵袭和迁移^[14]^。同时研究^[15-18]^表明IL-23促进肿瘤微环境中Th0细胞分化为Th17细胞，Th17细胞产生IL-17A，进而招募包括巨噬细胞、中性粒细胞在内的炎性细胞浸润，分泌大量的炎性因子如MMP-2、MMP-9、IL-1、IL-6。而MMP-2、MMP-9是Ⅳ型胶原酶，它在肿瘤细胞突破基底膜屏障而发生浸润和转移的过程中起着重要作用^[19]^，提示IL-23间接地参与了肿瘤的侵袭与迁移，但IL-23对肿瘤的迁移和侵袭是否有直接的作用，目前尚未见报道。

本研究通过人肺腺癌组织HE染色发现在肿瘤细胞周围有大量的淋巴细胞浸润，说明肿瘤组织中有炎性反应，据此推测炎症与肺癌的发生、发展息息相关。同时本实验小组的另一项研究表明IL-23R在肺腺癌组织及A549细胞中都有表达（文章正在审稿中），因此我们进一步探讨IL-23是否可以通过IL-23R直接促进肿瘤细胞的迁移和侵袭。通过迁移和侵袭实验发现IL-23确实能够明显促进A549的迁移和侵袭，同时这一效应能完全为IL-23特异性的中和抗体所消除，说明IL-23具有直接促进A549细胞侵袭和迁移的作用。虽然有文献^[10]^报道IL-23有直接促进肿瘤细胞增殖的作用，我们的另一项研究也发现IL-23有直接促进肺腺癌细胞增殖的作用（文章正在审稿中）。但是我们的研究证实在10 ng/mL IL-23的作用下肺腺癌细A549的增殖相对PBS组增加不超过1倍，而本文研究发现A549细胞的迁移数增加了3倍以上，其迁移倍数远远超过增殖倍数，提示其迁移数的增加不完全是由增殖引起的，说明IL-23具有直接促进A549的侵袭及迁移的作用。

MMPs是与肿瘤发生相关的一类重要的蛋白酶，其相关的信号传导通路参与了肿瘤细胞的生长、肿瘤相关炎症的发生及肿瘤血管的形成^[20]^。目前发现的MMPs有23种，MMP-1^[21]^、MMP-7^[22]^、MMP-2、MMP-9^[23]^等与肿瘤的侵袭和迁移相关。其中巨噬细胞源性的MMP-2、MMP-9与肿瘤的炎性细胞侵润及转移关系尤为密切^[23]^。同时有研究^[24]^发现MMP-9在肿瘤新生血管的发生及转移灶的形成^[25]^中扮演着非常重要的角色。因此我们进一步研究了IL-23作用下MMP-9的表达，以探讨IL-23是否具有直接促进MMP-9产生的作用。定量PCR和ELISA的结果表明IL-23能上调*MMP-9*基因和蛋白的表达，其基因水平的表达在4 h达到最高峰，而蛋白水平的峰值在24 h后；同时该效应能被IL-23中和抗体所阻断，说明IL-23有直接促进MMP-9表达的作用。因此，IL-23可能通过直接上调MMP-9表达来促进A549细胞的迁移和侵袭。

综上所述，IL-23不仅与肿瘤的发生和生长相关，还具有直接促进肿瘤转移的作用，因此它可能会成为一个新的生物治疗靶点而应用于临床。
